# Predictive factors for treatment outcomes with intravitreal anti-vascular endothelial growth factor injections in diabetic macular edema in clinical practice

**DOI:** 10.1186/s40942-023-00453-0

**Published:** 2023-04-04

**Authors:** Rajya L Gurung, Liesel M FitzGerald, Ebony Liu, Bennet J McComish, Georgia Kaidonis, Bronwyn Ridge, Alex W Hewitt, Brendan J Vote, Nitin Verma, Jamie E Craig, Kathryn P Burdon

**Affiliations:** 1 Menzies Institute for Medical Research, University of Tasmania, 17 Liverpool Street (Private Bag 23), Hobart, TAS 7000 Australia; 2 Department of Ophthalmology, Flinders Health and Medical Research Institute, Flinders University, Adelaide, South Australia; 3 School of Medicine, University of Tasmania, Hobart, TAS Australia

**Keywords:** Anti-VEGF, Diabetic macular edema, Best-corrected visual acuity, Central macular thickness

## Abstract

**Background:**

Intravitreal anti-vascular endothelial growth factor (anti-VEGF) injections are the standard of care for diabetic macular edema (DME), a common complication of diabetes. This study aimed to identify factors influencing DME intravitreal anti-VEGF treatment outcomes in real-world practice.

**Methods:**

This was a multi-center retrospective observational study using medical chart review of participants receiving anti-VEGF injections for DME (N = 248). Demographic and clinical variables were assessed for association with best corrected visual acuity (BCVA) and central macular thickness (CMT) outcomes using regression models.

**Results:**

There was a significant improvement in BCVA (p < 0.001) and CMT (p < 0.001) after 12 months of treatment, although 21% of participants had decreased BCVA, and 41% had a < 10% CMT reduction at 12 months. Higher baseline BCVA (p = 0.022, OR=-0.024, 95% CI=-0.046,-0.004) and longer duration of diabetic retinopathy (p = 0.048, OR=-0.064, 95% CI=-0.129,-0.001) were negative predictors for BCVA response, whereas Aflibercept treatment (p = 0.017, OR = 1.107, 95% CI = 0.220,2.051) compared with other drugs and a positive “early functional response” (p < 0.001, OR=-1.393, 95% CI=-1.946,-0.857) were positive predictors. A higher baseline CMT (p < 0.001, OR = 0.019, 95% CI = 0.012,0.0261) and an “early anatomical response”, (p < 0.001, OR=-1.677, 95% CI=-2.456, -0.943) were predictors for greater reduction in CMT. Overall, the variables could predict only 23% of BCVA and 52% of CMT response.

**Conclusions:**

The study shows a significant proportion of DME patients do not respond to anti-VEGF therapy and identifies several clinical predictors for treatment outcomes.

**Trial registration:**

The study was approved through the Human Research Ethics Committee, University of Tasmania (approval number H0012902), and the Southern Adelaide Clinical Human Research Ethics Committee (approval number 86 − 067).

**Supplementary Information:**

The online version contains supplementary material available at 10.1186/s40942-023-00453-0.

## Introduction

Diabetic macular edema (DME) is the leading cause of vision loss in the working-age population [1, 2]. Intra-ocular anti-VEGF therapies (the gold standard treatment for DME) clearly show benefits over the previous standard of care (laser therapy), however, it is clear that they are not effective for all DME patients [3–5]. Investigating possible predictors related to treatment efficacy, is essential for further understanding, prognosis prediction, and personalized treatment of DME. Multiple studies have found baseline vision prior to treatment, baseline central macular thickness (CMT), and undertreatment, to be the most significant indicators of anti-VEGF response [6–8]. Better baseline visual acuity is associated with superior final visual outcomes; however, in eyes with good baseline visual acuity, there is typically a smaller increase in visual acuity due to a ‘ceiling effect’ where good vision is reached and further improvements cannot be made. On the contrary, poor baseline visual acuity is predictive of a larger increase in vision [9]. For similar reasons, lower baseline CMT (thinner macula) has been shown to have superior anatomical outcomes but smaller overall reduction in CMT [10–13]. A growing number of studies today have attempted to use spectral domain optical coherence tomography based (SD-OCT based) imaging biomarkers for diagnosis, monitoring and treatment-prediction of DME. The biomarkers include retinal hyper-reflective foci [14–19], presence of disorganization of the inner retinal layers [20] [21, 22], disruption of ellipsoid zone [12, 23–25], disruption of external limiting membrane [13, 23, 26, 27], vitreo-macular status [28], and intra-retinal cyst [29]. Response to anti-VEGF injections has also been reported to be influenced by different morphological subtypes of DME, with the sub-type diffuse retinal thickening showing the least improvement and serous retinal detachment showing the most improvement [30]. Diabetic macular ischemia (characterized by increased foveal avascular zone), which often occurs alongside DME also contribute to poor treatment outcome [31, 32]. While fluorescein angiography (FA), an invasive imaging modality is commonly used to evaluate FAZ [33], more recently FA is being replaced by OCT angiography (OCT-A), a non-invasive imaging technology [34, 35].

Most studies on predictors of treatment response are from post-hoc analysis of randomized trials [6, 7, 9, 10, 26, 36–41], with limited studies from real-world practice exclusively designed to examine the prognostic factors [11, 12, 42]. The majority of the real-world studies were retrospective [11, 43], performed at a single medical center [20, 23], had limited sample size (sample size as low as 15 eyes) [28, 32], and limited follow-up [23, 32]. An additional issue with these studies is the lack of consistency in results across studies, such as hyper-reflective foci being a favourable predictor by some [43, 44] and a negative predictor by others [45, 46]. Some of the largest real-world studies published so far have evaluated outcomes of anti-VEGF injections rather than looking at predictors [47–49]. Consequently, there remains a significant unmet need for real-world studies to explore the possible clinical predictors of treatment response. The aim of the present study was, therefore, to assess outcome and the predictors of treatment response to anti-VEGF injections in routine clinical practice.

## Materials and methods

### Study design

This was a retrospective observational study based on detailed medical chart review of participants enrolled through the Tasmanian Ophthalmic Biobank (University of Tasmania in collaboration with local eye clinics in Tasmania) and the Genetic Risk Factors in Complications of Diabetes study (Flinders University, South Australia). Both studies adhered to the tenets of the Declaration of Helsinki in accordance with the relevant ethics guidelines.

### Study participants and data collection

The cohort and data acquisition have been described previously [50]. Briefly, the study included Type 1 (T1) or Type 2 (T2) diabetes patients (≥ 18 years) who received intravitreal anti-VEGF injections (Bevacizumab; Genentech: Ranibizumab; Novartis: Aflibercept, Regeneron) between 2013 and 2020 for the treatment of CI-DME confirmed by optical coherence tomography (OCT). Patients were excluded if they had any of the following conditions within six months prior to the first injection: systemic anti-VEGF therapy, intra-ocular steroid, vitreoretinal surgery, severe media opacity obscuring detailed fundus evaluation, and/or follow-up data for less than 12 months. In patients receiving bilateral anti-VEGF injections, the better-responding eye was chosen for the study. Relevant demographic and ocular parameters were retrospectively collected from medical charts as previously described [50]. BCVA in Snellen’s visual acuity score was converted to early treatment diabetic retinopathy study (ETDRS) letter scores [51].

### Outcome measures

The primary outcome measures were change in BCVA (functional outcome), measured as ETDRS letter scores, and change in central macular thickness (CMT; anatomical outcome), measured by OCT 12 months after the first intravitreal anti-VEGF injection. For the functional outcome, we categorized participants as: “good responders” - improvement of 5 ETDRS letters or more from the baseline, “moderate responders” − 0 to < 5 ETDRS letters improvement from baseline, and “poor-responders” - any loss of vision from baseline. An anatomical responder was defined as a 10% or greater reduction in CMT from baseline. The secondary outcomes were the mean change in BCVA and CMT at four months to determine if early response could predict later outcomes. An “early functional response” was defined as an improvement of 5 ETDRS letters or more from the baseline at four months, while “early anatomical response” was defined as a 10% or greater reduction in CMT from the baseline at four months. Next, we also assessed the proportion of individuals who experienced combined functional (≥ 5 ETDRS letters improvement) and anatomical response (≥ 10% CMT reduction).

### Statistical analysis

Results are presented as the mean ± standard deviation (SD) for continuous variables and as proportions (%) for categorical variables. The normality of all quantitative variables was assessed by visualizing the Q − Q plot and histogram outputs, and parametric or non-parametric tests were applied where applicable. Wilcoxon-signed rank test was used to compare final vision and final CMT with baseline values across all participants. For the functional outcome, between-group analyses of the three levels of outcome were performed using the Kruskal-Wallis H-test for continuous variables and the Chi-square test for categorical variables. For anatomical outcome with two categories, the Mann-Whitney U test for continuous variables and Chi-square test for categorical variables were used.

To identify predictors of functional response, ordinal logistic regression models were used with covariates (baseline BCVA, baseline CMT, number of injections, early functional responder, early anatomical responder, injection type, duration of DR, DME subtype, lens status, laterality, age, sex, smoker status, nephropathy, hyperlipidemia, HTN, body mass index (BMI), DM duration, HbA1c, DM type, anti-VEGF drug type) included in the multivariable model. For the binary anatomical response, binary logistic regression was used, incorporating the same covariates in the model. For all analyses, covariates sex (male:female), current or past smoking status (yes:no), HTN (yes:no), hyperlipidemia (yes:no), nephropathy (yes:no), PRP at baseline (yes:no), focal laser at baseline (yes:no), laterality of eye (R/L), lens status (phakic:pseudophakic), DM type (T1:T2), drug type (Insulin = yes:no), early functional responder (yes:no), and early anatomical responder (yes:no) were dichotomized. Multivariable analyses used variables that showed statistical significance in univariable analyses and those reported in previous studies. The Nagalkerke R-square statistic (R^2^) from the regression analysis was reported as a measure of the proportion of variability in the different categories of outcomes that was explained by the variables included in the model. We assessed the correlations between “BCVA and CMT changes” from baseline to “month four” and those from baseline to “month 12” using the Pearson’s correlation test. The relationship between change in BCVA and CMT at 12 months was also assessed by Pearson correlation coefficient. Statistical analyses and data visualization were performed using R version 4.0.2 (http://www.R-project.org/). A p-value of < 0.05 was considered statistically significant.

## Results

### Overall baseline clinical characteristics and treatment received by the participants

A total of 248 participants were included in the study (Table 1). The mean age was 66.92 ± 12.19 years. More than half (64.91%) of participants were male, and on average participants had high BMI (33.56 ± 7.85 kg/m2), long duration of diabetes (22.44 ± 9.96 years) and DR (8.06 ± 4.23 years), and poorly controlled DM (HbA1c = 8.32 ± 1.62 %). The majority of participants (84.67%) had T2DM with a high proportion of comorbid conditions, including HTN (85.88%) and hyperlipidemia (90.72%). Just over half of the participants had concomitant renal dysfunction (Nephropathy = 55.24%). Over two-thirds of participants had received laser therapy [pan-retinal photocoagulation (PRP = 42.74%) or focal laser (39.51%)] for DR at the time of enrolment. The cumulative mean injection number at the end of 12 months was 8.06, with over half of patients (55.64%) receiving Bevacizumab as the anti-VEGF injection of choice. No adverse events post anti-VEGF injections were recorded.

**Table 1:** Overall baseline clinical characteristics and treatment received

Variable		N = 248
**Patient related**		**Parameter**
Age (years)		66.92 (12.19)
BMI (kg/m^2^)		33.56 (7.85)
DM duration (years)		22.44 (9.96)
HbA1c %		8.32 (1.62)
Gender:Male		161 (64.91%)
Smoker:Yes		127 (51.20%)
DM:T2		210 (84.67%)
Drug:Insulin		182 (73.38%)
HTN:Yes		213 (85.88%)
Nephropathy:Yes		137 (55.24%)
Hyperlipidemia:Yes		225 (90.72%)
**Eye related**		
Baseline BCVA (ETDRS letters)		63.63 (14.93)
Baseline CMT (microns)		381.64 (107.31)
DR duration (years)		8.06 (4.23)
Laterality: RE		123 (49.59%)
Lens status: Phakic		164 (66.12%)
PRP:Yes		106 (42.74%)
Focal:Yes		98 (39.51%)
*DR severity*		
	Mild NPDR	53 (21.37%)
	Moderate NPDR	74 (29.83%)
	Severe NPDR	40 (16.12%)
	PDR	81 (32.66%)
*Drug received*		
	Bevacizumab	138 (55.64%)
	Aflibercept	31 (12.50%)
	Ranibizumab	45 (18.14%)
	Mixed	34 (13.70%)

Abbreviations: BCVA = best corrected visual acuity; BMI = body mass index; CMT = central macular thickness; DM = diabetes mellitus; DR = diabetic retinopathy; ETDRS = early treatment diabetic retinopathy study; HTN = hypertension; NPDR = non-proliferative diabetic retinopathy; PDR = proliferative diabetic retinopathy; PRP = pan-retinal photocoagulation

Data are presented as means (SD) for continuous variables and number (percentage) for categorical variables.

### Outcome measures at different time points

**Fig. 1:**
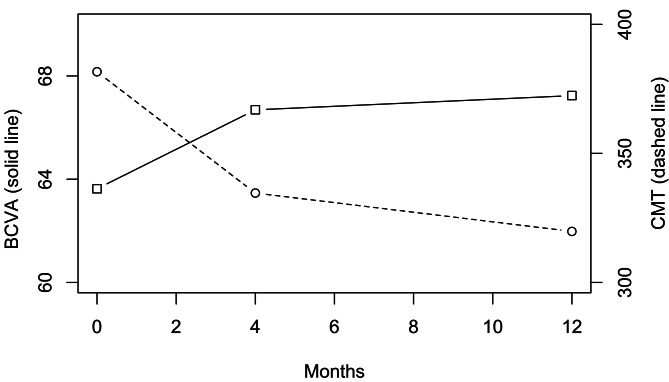
Mean outcome measures at baseline, 4 months and 12 months

Both BCVA and CMT improved over 12 months of treatment (Fig. 1). There was a statistically significant improvement in BCVA (p < 0.001) and a reduction in CMT (p < 0.001) at the end of 12 months with a mean improvement in BCVA of 3.6 ETDRS letters (± 10.99), and a mean reduction in CMT of 61.85 microns (± 103.80). Similarly, within the first four months, there was significant change in BCVA (p < 0.001) and CMT (p < 0.001), with mean improvement of BCVA of 3.16 ETDRS letters (± 9.86) and mean CMT reduction of 46.99 microns (± 91.33). There was a significant positive correlation between change in BCVA at four months and at 12 months (correlation coefficient = 0.596, p < 0.001, 95% CI = 0.50, 0.67), as well as between CMT measures at four and 12 months (correlation coefficient = 0.81, p < 0.001, 95% CI = 0.76, 0.85). There was statistically significant negative correlation between absolute changes in BCVA and CMT at 12 months, i.e., increase in BCVA was associated with a decrease in CMT (Correlation coefficient=-0.30, p < 0.001, 95% CI=-0.413, -0.187).

Left-hand-axis represents mean best-corrected visual acuity (BCVA) in ETDRS letters. The right-hand-axis represents mean central macular thickness (CMT) in microns.

### Baseline characteristics of patients in each 12 months response category

For functional outcome, after 12 months of treatment 51.61% of the cohort were good responders (≥ 5 ETDRS letters improvement), 27.41% were moderate responders (0 to < 5) ETDRS letters improvement), and 20.96% were poor responders (loss of vision from baseline). The three groups differed significantly in their baseline BCVA (p < 0.001) (Supplementary Table 1). Pairwise comparison revealed a statistically significant difference between good responders and poor responders (p = 0.001) and between good responders and moderate responders (p < 0.001). There was no significant difference in baseline BCVA between the poor and moderate responders. The three groups were comparable in the rest of their baseline and clinical characteristics. Only 14.11% (35/248) had ≥ 15 ETDRS letters improvement at the end of 12 months.

For anatomical outcome, at the end of 12 months, 59.27% were good responders (≥ 10% CMT reduction) and 40.72% were poor responders (< 10% CMT reduction). The two groups were comparable in all their baseline characteristics except for BMI, HbA1c and CMT (Supplementary Table 2). Both the BMI and baseline HbA1c were significantly higher for the poor responder group (p < 0.05), whereas baseline CMT was significantly higher for the good responders (p < 0.001).

Of the total participants, 31.85% (N = 79) showed combined response (≥ 5 ETDRS letters improvement and ≥ 10% CMT reduction) while 20.96% (N = 52) showed combined non-response (< 5 ETDRS letters improvement and < 10% CMT reduction), and 47.17% (N = 117) improved in a single criterion i.e., either BCVA or CMT.

For both the functional and anatomical criteria, the brand of drug received was comparable between the various response categories (p > 0.05).

### Predictors of treatment outcomes

**Fig. 2:**
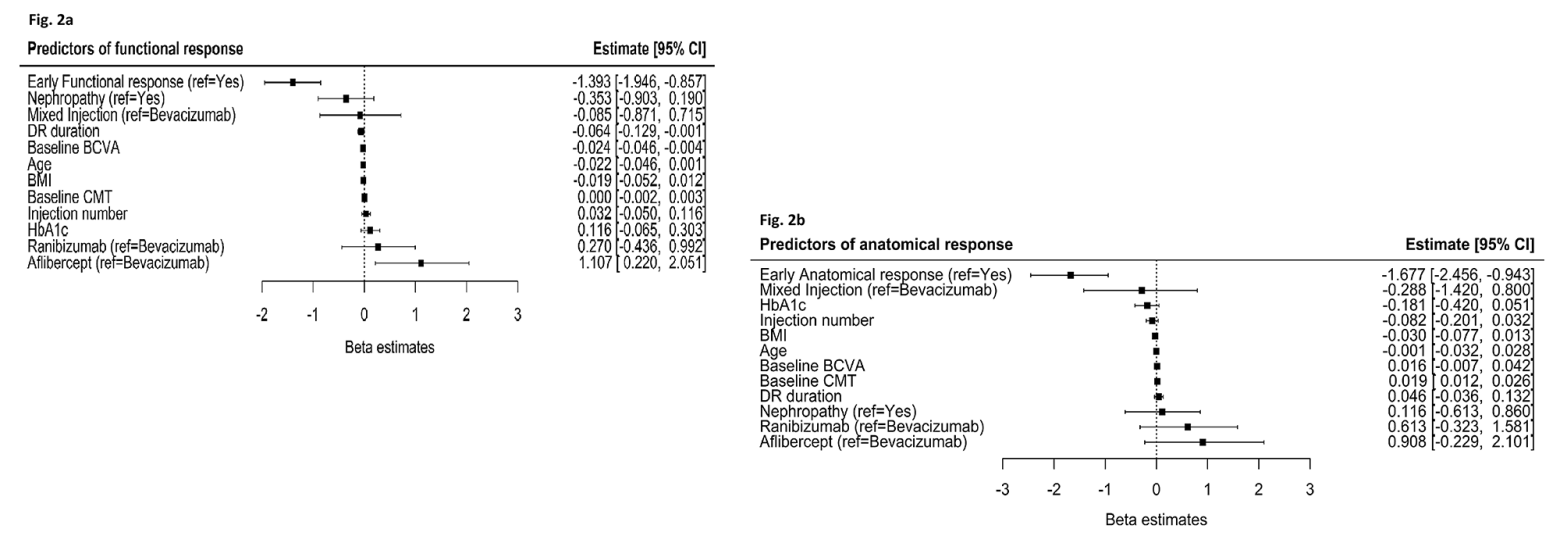
Predictors of response (Multivariable regression) at the end of 12 months: 2a Functional response; 2b Anatomical response

The results of ordinal logistic regression investigating the predictors of functional outcome are summarized in Fig. 2a and Supplementary Table 3. Patients with a higher baseline BCVA were less likely to be categorized as good responders under a univariable (p = 0.008) and multivariable model (p = 0.022). An inability to achieve “early functional response,” defined as ≥ 5 ETDRS letters improvement at four months, was negatively associated with a good final response in both the univariable (p < 0.001) and multivariable (p < 0.001) models. Moreover, the likelihood of being categorized as a good responder (compared with moderate or poor responder) was significantly higher for those who received Aflibercept as opposed to Bevacizumab for both univariable (p = 0.042) and multivariable models (p = 0.017), and likewise Aflibercept as opposed to Mixed injection (p = 0.038, multivariable). There was also evidence that a long duration of DR was associated with poor response under both univariable and multivariable models (p = 0.018 and p = 0.048, respectively). This ordinal regression model explained 23.09% (Nagelkerke R) of the variance in visual outcome.

The results of binary logistic regression exploring the predictors of anatomical outcome are given in Fig. 2b and Supplementary Table 4. For both univariable and multivariable models, baseline CMT was positively associated with a good anatomical response (p < 0.001). Similar to functional outcome above, failure to achieve an “early anatomical response,” defined as ≥ 10% CMT reduction at four months, was negatively associated with a good anatomical response (p < 0.001). Under a univariable model, there was also evidence that a higher HbA1c was associated with a poor response (p = 0.018); however, this result was not significant once other covariates were adjusted for (p = 0.129). The regression model explained 52.33% (Nagelkerke R) of the variance in ≥ 10% CMT reduction.

The dashed vertical line represents the point of no effect (null hypothesis). The black boxes represent the point estimates and the error bars represent the corresponding 95% confidence intervals (CIs). BCVA = best-corrected visual acuity; BMI = body mass index; CMT = central macular thickness; DR = diabetic retinopath.

## Discussion

This study shows that DME patients treated with anti-VEGF injections have variable treatment outcomes in a real-world setting. There was a significant improvement in both BCVA (functional outcome) and CMT (anatomical outcome) after 12 months of treatment, with 14.11% showing ≥ 15 ETDRS letters improvement (equivalent to three lines of Snellen visual acuity). However, a significant proportion of patients (20.96%) lost vision and an even higher proportion (40.72%) had poor anatomical outcomes. Higher baseline BCVA and longer DR duration were negative predictors of functional response, whereas Aflibercept treatment and an early functional response were positive predictors for vision improvement. For anatomical response, a higher baseline CMT and an “early anatomical response” were predictors for greater reduction in CMT, while baseline HbA1c value was a negative predictor for reduction in CMT.

DME patients in this study showed less overall vision improvement than RCTs testing the effectiveness of anti-VEGF therapy. Trials for DME reported 9.7–13.3 ETDRS letters gain [3, 52, 53] compared with only three letters on average in the current study. For example, in the DRCR.net Protocol I, there was a ≥ 5 ETDRS letters improvement in 68–76% of participants in the Ranibizumab groups [52], while in the RESTORE study, there was ≥ 5 ETDRS letters improvement in 63.3–65.2% of participants in the Ranibizumab groups at the end of 12 months [54]. However, in our study, only half of the participants (51.61%) had a ≥ 5 ETDRS letters improvement, with ~ 20% of participants losing vision at final follow-up. Our findings are consistent with other real-world observational studies, showing that patients with DME have lower visual gains than in clinical trials [55–57]. Poor treatment outcome has been attributed to undertreatment in many real-world studies [47, 49]. However, the mean injection number (8.06) for this study was better than reported by other real-world studies [49]. Despite a relatively high number of injections, the mean improvement in vision (3.06 ETDRS letters) was much lower than in RCTs; the exact reason for this remains unknown, though another study from Australia also reports similar findings (mean improvement of 4.31 ETDRS letters, mean injection number = 11.2) [58]. A possible explanation for this suboptimal outcome observed in our study might be attributed to the “ceiling effect” as discussed below.

For functional outcome, the good responder group had significantly lower baseline vision (61.08 ETDRS letters) compared with the other two groups. Poor baseline vision has previously been associated with greater vision improvements [10, 59]. A ceiling effect, where there is no further room for improvement in people with better starting vision may be an explanation for this finding [60]. However, both the poor responder and moderate responder groups had baseline BCVA < 69 ETDRS letters, providing sufficient room for improvement and suggesting other factors may be at play. Also, to note that a significant proportion of our participants (54.56%) with poor baseline vision (< 58 ETDRS letters) continued to suffer from poor final vision, despite treatment and despite having enough room for improvement; further evidence that ceiling effect may not be the reason for sub-optimal outcome.

Another important predictor of visual response in this study was the type of anti-VEGF injection administered. The injection subtype, Aflibercept, was positively associated with good response after adjusting for other confounding factors, including baseline BCVA. Although this finding is uncertain as it is based on small numbers, it is consistent with DRCR.net (Protocol T), which showed Aflibercept had a clear advantage over Bevacizumab or Ranibizumab at one year of follow-up for DME patients with vision 69 ETDRS letters or worse [61]. Likewise, a slightly higher efficacy of Aflibercept injection was observed in a Cochrane meta-analysis of 24 studies with 6007 patients in total [62]. Interestingly, the mean baseline BCVA of all three groups in our study was < 69 ETDRS letters (similar to the sub-group in Protocol T which showed better response with Aflibercept); hence this might explain the greater efficacy of Aflibercept injection in our study cohort. However, this finding should be interpreted with caution as our study had only 31 participants in the Aflibercept injection group. Thus, larger Aflibercept cohorts should be analyzed to validate our findings, though a recently published real-world study by Bhandari et al. corroborates our finding [63]. Their study compared 12-month treatment outcomes of Ranibizumab and Aflibercept in routine clinical practice using a relatively large cohort of 383 eyes (Ranibizumab = 166 eyes, Aflibercept = 217 eyes). Larger visual gains and CMT reductions were achieved in the Aflibercept group [63]. Interestingly, in the comparative analysis mentioned above (DRCR.net, Protocol T), the greater visual benefit of Aflibercept over Ranibizumab or Bevacizumab at the end of two years was deemed clinically doubtful [61]. Another real world study by Huang et al. [18] also showed comparable visual outcomes between Ranibizumab and Aflibercept through one year of follow up. Therefore, the difference in outcomes cannot be directly attributed to differences in anti-VEGF agents. Additional RCTs (with fixed identical treatment protocol across all the three agents) with a longer follow up duration are warranted to assess the generalizability of our findings.

Next, an “early functional response at four months” was a significant predictor of long-term visual outcome (12 months) in this study. There was a positive correlation between BCVA at four months and final BCVA at 12 months, and early responders were more likely to be categorized as good responders at 12 months. Similar observations were made in a post-hoc analysis of DRCR.net (Protocol I), where eyes with a suboptimal early BCVA response (< 5 ETDRS letters improvement at three months) showed poorer long-term visual outcomes than eyes with a positive early response [64]. A real-world study by Koyanagi et al. [65] also confirmed that an early response predicted visual outcome at 12 months in DME patients treated with anti-VEGF drugs. Early indicators of long-term vision outcomes are valuable to ophthalmologists and patients alike as they can inform decisions around patient counseling and monitoring. Based on our findings, it would be tempting to recommend a change in the treatment regimen for individuals with poor early outcome (four months); however, other studies have shown that an early sub-optimal response does not always preclude long term outcome [66] [67]. In the posthoc analysis of DRCR.net (Protocol T), eyes with less than 5-letter gain at three months often had good vision at two years without switching therapies [66]. This report showed good visual gain at two years in many eyes despite limited initial response at three months. Specifically, among eyes with early poor response (< 5 ETDRS letters improvement at three months), the percentage of eyes gaining 10 or more additional letters from three months at two years was 38% (18 of 48) with Aflibercept, 38% (26 of 68) with Bevacizumab, and 42% (25 of 59) with Ranibizumab [66]. This was further supported by another post-hoc report which showed continued vision gains in many eyes despite limited initial response and persistent sub-retinal fluid [67]. On the contrary, many studies report better outcomes when switched to alternative therapy at an earlier time point [68, 69]. A study by Hernandez Martinez et al. [68] compared the effects of dexamethasone implant on functional and anatomical outcomes in patients switched to steroid therapy following poor response to anti-VEGF therapy. In this study, eyes switched to early steroid (after three anti-VEGF) obtained better functional and anatomical outcomes than those who underwent later switch (after six injections) [68]. Additionally, early switch was associated with a cost saving of € 3057.8 as reported by Ruiz-Moreno et al. [70]. Ultimately it remains unknown at this time, whether alternative therapies would benefit eyes with limited initial response, hence the need for future studies and meta-analyses to explore this further.

Duration of DR was a negative predictor of good visual response in this study. This may be due to the fact that a longer duration of disease produces ongoing macular damage, causing irreversible vision loss. This is likely explained by photoreceptor and ganglion cell damage, a consequence of long-standing macular fluid [71]. Further, a longer duration of DR may signify a transition from an acute inflammatory phase to a more difficult to treat, chronic inflammation phase [38, 67]. Previous studies have demonstrated that an early DME diagnosis offers the opportunity for prompt anti-VEGF treatment and the prospect of a more favorable outcome than if treatment was delayed [3, 72]. Prior studies have found smaller gains in vision with longer duration of diabetes [7, 10] however, the association between diabetes duration and visual outcomes was not confirmed in our study. Instead, duration of DR may be a better predictor of response as seen in this study. A similar observation of no association with diabetes duration but worse visual outcome with long duration of DME was made by Lee et al. [73]. Despite a long duration of diabetes, patients may still have good glycemic control and be at low risk of developing diabetes-related complications, such as DME [74].

Anatomically, baseline CMT was negatively associated with reduction in CMT in this study, as reported in previous studies [12, 42]. Baseline CMT has been shown to be one of the strongest predictors of anatomical outcome. Further, reduction in CMT during the first treatment year has been associated with better long-term visual outcomes [75]. Similar to the functional analysis, a positive early response was identified as a predictor for greater reduction in CMT at 12 months, a finding which is corroborated by other studies [12, 42].

A significant finding from this study is that despite including a large number of potential clinical and ocular predictors, only 23.09% of functional response and 52.33% of anatomical response could be explained by the predictors. This suggests a large portion of treatment response is unaccounted for by conventional risk factors. Notably, a similar finding was reported by the Wisconsin Epidemiologic Study of Diabetic Retinopathy (WESDR) [76] and the Diabetes Control and Complications Trial (DCCT) [77], where conventional risk factors explained only a small fraction of the risk for DR development (10%) and progression (15%). It is therefore clear that other clinical, demographic, genetic and/or epigenetic factors are involved. Studies exploring such potential risk factors are required and are beginning to emerge [78, 79]. Further, less than a third of DME patients (31.85%) showed combined response (functional and anatomical) with a weak negative correlation (Correlation coefficient =-0.30) observed between the two outcome categories. This relationship between BCVA and CMT is poorly understood with most studies reporting a weak to moderate correlation between the two [80, 81].

The biggest limitation of this study would be the unavailability of detailed OCT data. When evaluating OCT, we should be mindful that CMT is not the only parameter. Other important parameters and morphological characteristics in OCT (e.g.: disorganization of retinal inner layers; inner segment-outer segment integrity; hyper-reflective retinal foci; disruption of external limiting membrane) could provide greater and better insights into treatment outcomes [82]. A more comprehensive analysis of these factors was not possible due to lack of relevant data in the retrospective study design. Apart from OCT, other imaging modalities like FA and more recently OCT-A can be incorporated in evaluation of DME [83, 84]. Both these imaging modalities especially help in assessment of DME including, macular microvasculature, foveal avascular zone, and ischemic maculopathy; factors which need to be considered when assessing poor treatment outcomes in DME [32, 85]. FA while is considered an invasive procedure was not performed in majority of our study participants, and OCT-A (a non-invasive modality) was not available in any of the centers at the time of data collection.

Further, no single type of anti-VEGF agent was used consistently in our patient cohorts, who were treated at the clinician’s discretion. Across the disease cohorts, many patients received two or three different anti-VEGF agents over the course of 12 months; however, this reflects real-world practices.

In conclusion, the results of this study confirm that DME patients receiving anti-VEGF therapy in routine clinical practice achieve inferior outcomes to patients in landmark clinical trials. A small but significant number of patients continue to lose vision, despite repeated anti-VEGF injections. This has significant implications for our clinical management of DME patients. Other treatment options, including intravitreal corticosteroids, laser photocoagulation, or surgical intervention, may be warranted for patients who lose vision despite repeated anti-VEGF therapy and consideration of these based on early response may be appropriate in high-risk individuals. Further, though this study offers useful clinical insights into the possible predictors of treatment outcome, future studies should aim to explore predictors beyond the conventional clinical and demographic risk factors.

## Electronic supplementary material

Below is the link to the electronic supplementary material.

Supplementary Material 1

## Data Availability

Data are available from the corresponding author upon reasonable request.
