# Supplementary material for: Predictive factors for treatment outcomes with intravitreal anti-vascular endothelial growth factor injections in diabetic macular edema in clinical practice
Source: Int J Retina Vitreous. 2023 Apr 4;9:23. doi: 10.1186/s40942-023-00453-0 (PMC10074667; doi:10.1186/s40942-023-00453-0)
Supplement: Supplementary file 1 — Supplementary Material 1 [file 40942_2023_453_MOESM1_ESM.docx]

| **Supplementary Table 1: Baseline and clinical characteristics of patients in each level of functional response** | | | | | |
| --- | --- | --- | --- | --- | --- |
|  | | **Poor responder**  **(N=52/20.96%)** | **Moderate responder**  **(N=68/27.41%)** | **Good responder**  **(N=128/51.61%)** | **P value*** |
| ***Individual related*** | | | | | |
| Age (years) | | 69.13 (10.03) | 67.54 (12.06) | 65.68 (12.96) | 0.463 |
| BMI (kg/m^2^) | | 33.98 (6.62) | 33.86 (9.18) | 33.23 (7.58) | 0.528 |
| DM duration (years) | | 22.77 (9.42) | 24.24 (9.39) | 21.35 (10.39) | 0.140 |
| HbA1c (g/dl) | | 8.02 (1.44) | 8.34 (1.68) | 8.43 (1.64) | 0.232 |
| Gender: Male | | 33 (63.46%) | 40 (58.82%) | 88 (68.75%) | 0.373 |
| Smoker: Yes | | 23 (44.23%) | 29 (42.64%) | 54 (42.18%) | 0.969 |
| DM: T2 | | 44 (84.61%) | 57 (83.82%) | 109 (85.15%) | 0.970 |
| Drug: Insulin | | 36 (69.23%) | 49 (72.05%) | 97 (75.78%) | 0.641 |
| HTN: Yes | | 46 (88.46%) | 59 (86.76%) | 108 (84.37%) | 0.749 |
| Nephropathy: Yes | | 27 (51.92%) | 40 (58.82%) | 70 (54.68%) | 0.740 |
| Hyperlipidemia: Yes | | 49 (94.23%) | 61 (89.70%) | 115 (89.84%) | 0.588 |
| ***Eye related*** | | | | | |
| Baseline BCVA  (ETDRS letters) | | 67.29 (13.23) | 65.65 (17.30) | 61.08 (13.83) | **<0.001^†^** |
| Baseline CMT (microns) | | 362.98 (97.96) | 383.31 (125.12) | 388.33 (100.46) | 0.133 |
| DR duration (years) | | 9.13 (4.19) | 8.32 (4.62) | 7.49 (3.95) | 0.070 |
| Laterality: RE | | 29 (55.76%) | 27 (39.70%) | 67 (52.34%) | 0.145 |
| Lens status: Phakic | | 32 (61.53%) | 43 (63.23%) | 89 (69.53%) | 0.495 |
| PRP: Yes | | 23 (44.23%) | 29 (42.64%) | 54 (42.18%) | 0.969 |
| Focal laser: Yes | | 20 (38.46%) | 26 (38.23%) | 52 (40.62%) | 0.934 |
| *DR severity* | | | | | 0.962 |
|  | Mild | 9 (17.30%) | 17 (25.00%) | 27 (21.09%) |  |
|  | Moderate | 18 (34.61%) | 19 (27.94%) | 37 (28.90%) |  |
|  | Severe | 8 (15.38%) | 11 (16.17%) | 21 (16.40%) |  |
|  | PDR | 17 (32.69%) | 21 (30.88%) | 43 (33.59%) |  |
| *Drug received* | | | | | 0.305 |
|  | Bevacizumab | 34 (65.38%) | 38 (55.88%) | 66 (51.56%) |  |
|  | Ranibizumab | 6 (11.53%) | 16 (23.52%) | 23 (17.96%) |  |
|  | Aflibercept | 4 (7.69%) | 6 (8.82%) | 21 (16.40%) |  |
|  | Mixed | 8 (15.38%) | 8 (11.76%) | 18 (14.06%) |  |
| BCVA=best-corrected visual acuity; BMI=body mass index; CMT=central macular thickness; DM=diabetes mellitus; DME=diabetic macular edema; DR=diabetic retinopathy; ETDRS=early treatment diabetic retinopathy study; HTN=hypertension; PDR=proliferative diabetic retinopathy; PRP=pan-retinal photocoagulation;  Data are presented as means (SD) for continuous variables and number (percentage) for categorical variables. Kruskal-Wallis test for continuous variables and Chi-square test for categorical.  *p-values are for global test comparing all three responses. Significant p-values are bolded.  † poor-responder vs moderate responder p=1.000; **moderate responder vs good responder p<0.001;** **poor responder vs good responder p=0.001** | | | | | |

| **Supplementary Table 2: Baseline and clinical characteristics of patients in each level of anatomical response** | | | | |
| --- | --- | --- | --- | --- |
|  | | **Non-responder**  **(N=101/40.72%)** | **Responders**  **(N=147/59.27%)** | **P value** |
| Age | | 66.35 (12.01) | 67.31 (12.34) | 0.533 |
| BMI (kg/m^2^) | | 34.73 (7.98) | 32.75 (7.68) | **0.047** |
| DM duration (years) | | 23.90 (9.69) | 21.44 (10.05) | 0.054 |
| HbA1c (g/dl) | | 8.62 (1.62) | 8.12 (1.59) | **0.007** |
| Gender: Male | | 68 (67.32%) | 93 (63.26%) | 0.510 |
| Smoker: Yes | | 58 (57.42%) | 69 (46.93%) | 0.105 |
| DM: T2 | | 83 (82.17%) | 127 (86.39%) | 0.365 |
| Drug: Insulin | | 79 (78.21%) | 103 (70.06%) | 0.154 |
| HTN: Yes | | 85 (84.15%) | 128 (87.07%) | 0.517 |
| Nephropathy: Yes | | 62 (61.38%) | 75 (51.02%) | 0.107 |
| Hyperlipidemia: Yes | | 91 (90.09%) | 134 (91.15%) | 0.778 |
| ***Eye related*** | |  |  |  |
| Baseline BCVA (ETDRS letters) | | 64.55 (13.46) | 63.00 (15.88) | 0.705 |
| Baseline CMT (microns) | | 323.96 (55.04) | 421.27 (116.29) | **<0.001** |
| DR duration (years) | | 7.83 (4.21) | 8.22 (4.25) | 0.513 |
| Laterality: RE | | 47 (46.53%) | 76 (51.70%) | 0.424 |
| Lens status: Phakic | | 65 (64.35%) | 99 (67.34%) | 0.62 |
| PRP: Yes | | 45 (44.55%) | 61 (41.49%) | 0.632 |
| Focal laser: Yes | | 33 (32.67%) | 65 (44.21%) | 0.068 |
| *DR severity* | | | | 0.472 |
|  | Mild | 17 (16.83%) | 36 (24.48%) |  |
|  | Moderate | 30 (29.70%) | 44 (29.93%) |  |
|  | Severe | 17 (16.83%) | 23 (15.64%) |  |
|  | PDR | 37 (36.63%) | 44 (29.93%) |  |
| *Drug received* | | | | 0.164 |
|  | Bevacizumab | 59 (58.41%) | 79 (53.74%) |  |
|  | Ranibizumab | 15 (14.85%) | 30 (20.40%) |  |
|  | Aflibercept | 9 (8.91%) | 22 (14.96%) |  |
|  | Mixed | 18 (17.82%) | 16 (10.88%) |  |
| BCVA=best corrected visual acuity; BMI=body mass index; CMT=central macular thickness; DM=diabetes mellitus; DR=diabetic retinopathy; ETDRS=early treatment diabetic retinopathy study; HTN=hypertension; PRP=pan-retinal photocoagulation; PDR=proliferative diabetic retinopathy; RE=right eye; Data are presented as means (SD) for continuous variables and number (percentage) for categorical variables. Mann-Whitney U test for continuous variables and Chi-square test for categorical. Significant p-values are bolded. | | | | |

| **Supplementary Table 3.** **Univariable and multivariable ordinal logistic regression analyses investigating factors predictive of functional outcome** | | | | | | |
| --- | --- | --- | --- | --- | --- | --- |
| **Variables** | **Univariable** | | | **Multivariable** | | |
|  | ***B*** | **95% CI** | **P** | ***B*** | **95% CI** | **P** |
| Baseline BCVA (ETDRS letters) | -0.024 | -0.043, -0.007 | **0.008** | -0.024 | -0.046, -0.004 | **0.022** |
| Baseline CMT (microns) | 0.001 | -0.0007, 0.003 | 0.220 | 0.0005 | -0.002, 0.003 | 0.688 |
| Injection number | 0.0051 | -0.067, 0.088 | 0.892 | 0.032 | -0.050, 0.116 | 0.447 |
| Early functional response (Yes=ref) | -1.417 | -1.933, -0.916 | **<0.001** | -1.393 | -1.946, -0.857 | **<0.001** |
| Early anatomical responder (Yes=ref) | -0.171 | -0.652, 0.305 | 0.482 | - | - | - |
| *Injection type* |  |  |  |  |  |  |
| Aflibercept (Bevacizumab=ref) | 0.836 | 0.055, 1.683 | **0.042** | 1.107 | 0.220, 2.051 | **0.017** |
| Ranibizumab (Bevacizumab=ref) | 0.284 | -0.336, 0.921 | 0.374 | 0.270 | -0.436, 0.992 | 0.456 |
| Mixed (Bevacizumab=ref) | 0.164 | -0.548, 0.899 | 0.654 | -0.085 | -0.871, 0.715 | 0.831 |
| Ranibizumab (Aflibercept=ref) | -0.552 | -1.499, 0.351 | 0.239 | -0.836 | -1.868, 0.149 | 0.102 |
| Mixed (Aflibercept=ref) | -0.672 | -1.680, 0.303 | 0.182 | -1.193 | -2.343, -0.078 | **0.038** |
| Mixed (Ranibizumab=ref) | -0.119 | -0.965, 0.732 | 0.781 | -0.356 | -1.303, 0.589 | 0.459 |
| DR duration (years) | -0.067 | -0.123, -0.011 | **0.018** | -0.064 | -0.129, -0.0008 | **0.048** |
| *DR severity* |  |  |  |  |  |  |
| Moderate (Mild=ref) | -0.154 | -0.823, 0.508 | 0.649 | - | - | - |
| Severe (Mild=ref) | -0.006 | -0.781, 0.774 | 0.987 | - | - | - |
| PDR (Mild=ref) | -0.006 | -0.664, 0.645 | 0.984 | - | - | - |
| Severe (Moderate=ref) | 0.147 | -0.580, 0.888 | 0.692 | - | - | - |
| PDR (Moderate=ref) | 0.147 | -0.454, 0.750 | 0.630 | - | - | - |
| PDR (Severe=ref) | -0.000 | -0.730, 0.718 | 1.000 | - | - | - |
| PRP (Yes=ref) | 0.055 | -0.423, 0.532 | 0.820 | - | - | - |
| Focal (Yes=ref) | -0.084 | -0.571, 0.398 | 0.732 | - | - | - |
| Lens status (Phakic=ref) | -0.295 | -0.790, 0.201 | 0.243 | - | - | - |
| Laterality of eye (Right=ref) | -0.071 | 0.241, -0.295 | 0.767 | - | - | - |
| Age (years) | -0.017 | -0.038, 0.001 | 0.077 | -0.022 | -0.046, 0.0006 | 0.059 |
| Sex (Male=ref) | -0.266 | -0.756, 0.225 | 0.287 | - | - | - |
| Smoker (Yes=ref) | -0.077 | -0.550, 0.396 | 0.750 | - | - | - |
| Nephropathy (Yes=ref) | -0.016 | -0.491, 0.461 | 0.947 | -0.353 | -0.903, 0.190 | 0.204 |
| Hyperlipidemia (Yes=ref) | 0.302 | -0.500, 1.159 | 0.470 | - | - | - |
| Hypertension (Yes=ref) | -0.265 | -0.979, 0.415 | 0.452 | - | - | - |
| BMI (kg/m^2^) | -0.009 | -0.038, 0.019 | 0.515 | -0.019 | -0.052, 0.012 | 0.237 |
| DM duration (years) | -0.017 | -0.041, 0.006 | 0.151 | - | - | - |
| HbA1c (g/dl) | 0.103 | -0.042, 0.255 | 0.170 | 0.116 | -0.065, 0.303 | 0.215 |
| DM type (T1=ref) | 0.055 | -0.607, 0.700 | 0.868 | - | - | - |
| Drug type (Insulin=ref) | -0.255 | -0.784, 0.277 | 0.344 | - | - | - |
| Abbreviations: BCVA=best corrected visual acuity; BMI=body mass index; CI=confidence interval; DM=diabetes mellitus; DME=diabetic macular edema; DR=diabetic retinopathy; ETDRS=early treatment diabetic retinopathy study; HTN=hypertension; PRP=pan-retinal photocoagulation; ref=reference.  Adjusted for baseline BCVA, baseline CMT, early functional responder, injection number, injection type, age, nephropathy, BMI, HbA1c, DR duration; Significant p-values bolded. | | | | | | |

| **Supplementary Table 4.** **Univariable and multivariable binary logistic regression analyses investigating factors predictive of anatomical outcome** | | | | | | |
| --- | --- | --- | --- | --- | --- | --- |
| **Variables** | **Univariable** | | | **Multivariable** | | |
|  | ***B*** | **95% CI** | **P** | ***B*** | **95% CI** | **P** |
| Baseline BCVA (ETDRS letters) | -0.007 | -0.025, 0.009 | 0.421 | 0.016 | -0.007, 0.042 | 0.288 |
| Baseline CMT (microns) | 0.018 | 0.013, 0.024 | **<0.001** | 0.019 | 0.012, 0.026 | **<0.001** |
| Injection number | -0.008 | -0.092, 0.076 | 0.847 | -0.082 | -0.201, 0.032 | 0.164 |
| Early functional responder (Yes=ref) | -0.435 | -0.950, 0.073 | 0.095 | - | - | - |
| ≥Early anatomical responder (Yes=ref*)* | -2.289 | -2.963, -1.671 | **<0.001** | -1.677 | -2.456, -0.943 | **<0.001** |
| *Injection type* |  |  |  |  |  |  |
| Aflibercept (Bevacizumab=ref) | 0.601 | -0.216, 1.491 | 0.163 | 0.908 | -0.229, 2.101 | 0.124 |
| Ranibizumab (Bevacizumab=ref) | 0.401 | -0.292, 1.126 | 0.265 | 0.613 | -0.323, 1.581 | 0.204 |
| Mixed (Bevacizumab=ref) | -0.409 | -1.171, 0.343 | 0.286 | -0.288 | -1.420, 0.800 | 0.608 |
| Ranibizumab (Aflibercept=ref) | -0.200 | -1.219, 0.782 | 0.692 | -0.294 | -1.604, 0.981 | 0.653 |
| Mixed (Aflibercept=ref) | -1.011 | -2.071, -0.003 | *0.053* | -1.196 | -2.723, 0.258 | 0.113 |
| Mixed (Ranibizumab=ref) | -0.810 | -1.742, 0.096 | 0.082 | -0.902 | -2.239, 0.379 | 0.174 |
| DR duration (years) | 0.022 | -0.037, 0.083 | 0.472 | 0.046 | -0.036, 0.132 | 0.279 |
| *DR severity* |  |  |  | - | - | - |
| Moderate (Mild=ref) | -0.367 | -1.120, 0.366 | 0.330 | - | - | - |
| Severe (Mild=ref) | -0.448 | -1.307, 0.403 | 0.302 | - | - | - |
| PDR (Mild=ref) | -0.577 | -1.315, 0.137 | 0.118 | - | - | - |
| Severe (Moderate=ref) | -0.080 | -0.859, 0.706 | 0.839 | - | - | - |
| PDR (Moderate=ref) | -0.209 | -0.851, 0.426 | 0.519 | - | - | - |
| PDR (Severe=ref) | -0.129 | -0.902, 0.632 | 0.741 | - | - | - |
| PRP (Yes=ref) | 0.124 | -0.387, 0.636 | 0.633 | - | - | - |
| Focal (Yes=ref) | -0.490 | -1.025, 0.032 | *0.068* | - | - | - |
| Lens status (Phakic=ref) | -0.133 | -0.665, 0.403 | 0.625 | - | - | - |
| Laterality of eye (Right=ref) | -0.206 | -0.716, 0.300 | 0.424 | - | - | - |
| Age (years) | 0.006 | -0.014, 0.027 | 0.542 | -0.001 | -0.032, 0.028 | 0.905 |
| Sex (Male=ref) | 0.179 | -0.351, 0.718 | 0.510 | - | - | - |
| Smoker (Yes=ref) | 0.421 | -0.086, 0.935 | 0.105 | - | - | - |
| Nephropathy (Yes=ref) | 0.422 | -0.089, 0.941 | 0.108 | -0.116 | -0.860, 0.613 | 0.755 |
| Hyperlipidemia (Yes=ref) | -0.124 | -0.987, 0.765 | 0.778 | - | - | - |
| Hypertension (Yes=ref) | -0.237 | -0.956, 0.491 | 0.517 | - | - | - |
| BMI (kg/m^2^) | -0.032 | -0.065, 0.0001 | *0.053* | -0.030 | -0.077, 0.013 | 0.182 |
| DM duration (years) | -0.025 | -0.051, 0.0005 | *0.057* | - | - | - |
| HbA1c (g/dl) | -0.191 | -0.354, -0.034 | **0.018** | -0.181 | -0.420, 0.051 | 0.129 |
| DM type (T1=ref) | 0.320 | -0.381, 1.015 | 0.366 | - | - | - |
| Drug type (Insulin=ref) | 0.427 | -0.153, 1.030 | 0.155 | - | - | - |
| BCVA=best corrected visual acuity; CI=confidence interval; CMT=central macular thickness; DME=diabetic macular edema; DM=diabetes mellitus; DR=diabetic retinopathy; ETDRS=early treatment diabetic retinopathy study; HTN=hypertension; PRP=pan-retinal photocoagulation. Adjusted for baseline BCVA, baseline CMT, early functional responder, injection number, injection type, age, nephropathy, BMI, HbA1c, DR duration; Significant p-values bolded. | | | | | | |
